# Tissue Repair Mechanisms of Dental Pulp Stem Cells: A Comprehensive Review from Cutaneous Regeneration to Mucosal Healing

**DOI:** 10.3390/cimb47070509

**Published:** 2025-07-02

**Authors:** Jihui He, Jiao Fu, Ruoxuan Wang, Xiaojing Liu, Juming Yao, Wenbo Xing, Xinxin Wang, Yan He

**Affiliations:** 1 First Clinical College of the Ministry of Medicine, Wuhan University of Science and Technology, Wuhan 430064, China; hejh0521@wust.edu.cn (J.H.); jiaofu1009@163.com (J.F.); 13133410436@163.com (R.W.); lxjyouxiang8@163.com (X.L.); 19986450979@163.com (J.Y.); 13183206367@163.com (W.X.); xinxin_w417@163.com (X.W.); 2 Hubei Province Key Laboratory of Oral and Maxillofacial Development and Regeneration, Wuhan 430022, China

**Keywords:** chronic wounds, dental pulp stem cells, mucosa, scaffold materials, scarring

## Abstract

Repairing and regenerating tissue barriers is a key challenge in regenerative medicine. Stem cells play a crucial role in restoring the structural and functional integrity of key epithelial barrier surfaces, including the skin and mucosa. This review analyzes the role of dental pulp stem cells (DPSCs) and their derivatives, including extracellular vesicles, conditioned medium, and intracellular factors, in accelerating skin wound healing. The key mechanisms include: (1) DPSCs regulating inflammatory microenvironments by promoting anti-inflammatory M2 macrophage polarization; (2) DPSCs activating vascular endothelial growth factor (VEGF) to drive angiogenesis; (3) DPSCs optimizing extracellular matrix (ECM) spatial structure through matrix metalloproteinase/tissue inhibitor of metalloproteinase (MMP/TIMP) balance; and (4) DPSCs enhancing transforming growth factor-β (TGF-β) secretion to accelerate granulation tissue formation. Collectively, these processes promote wound healing. In addition, we explored potential factors that accelerate wound healing in DPSCs, such as oxidative stress, mechanical stimulation, hypertension, electrical stimulation, and organoid modeling. In addition to demonstrating the great potential of DPSCs for skin repair, this review explores their translational prospects in mucosal regenerative medicine. It covers the oral cavity, esophagus, colon, and fallopian tube. Some studies have found that combining DPSCs and their derivatives with drugs can significantly enhance their biological effects. By integrating insights from skin and mucosal models, this review offers novel ideas and strategies for treating chronic wounds, inflammatory bowel disease, and mucosal injuries. It also lays the foundation for connecting basic research results with clinical practice. This represents a significant step forward in tackling these complex medical challenges and lays a solid scientific foundation for developing more targeted and efficient regenerative therapies.

## 1. Introduction

Chronic wounds now affect over 6.5 million Americans, with annual treatment expenditures surpassing $25 billion, owing to increasing healthcare costs, an aging population, and the rising prevalence of diabetes and obesity [1,2]. This poses an enormous burden on society. The market for wound care products is up to $15.3 billion per year and up to $12 billion per year for scar treatment [3]. In patients with diabetes and obesity, the wound-healing process takes much longer. It is more expensive to leave a hypertrophic scar or keloid, which affects aesthetics and function and causes psychological and physical distress. This presents a significant challenge for dermatologists and plastic surgeons. Despite various treatment approaches, including gauze dressings, negative pressure wound therapy, and surgical methods, many lack robust efficacy [4].

Within the mucosal systems, the intestinal tract is one of the most prevalent sites of pathology. Ulcerative colitis (UC), a chronic relapsing-remitting inflammatory bowel disease localized to the colorectum, affected approximately 5 million individuals worldwide in 2023, with epidemiological data indicating a persistently rising incidence trend [5]. Clinical management adheres to a severity-stratified approach: mild cases typically receive first-line intervention with non-steroidal medications, while moderate-to-severe presentations require corticosteroids or biologic agents as primary therapies. Patients with corticosteroid refractoriness or dependency necessitate escalation to immunomodulatory regimens. Surgical intervention remains the definitive approach for end-stage cases or those with severe complications [6]. First-line UC pharmacotherapies (e.g., corticosteroids and sulfasalazine) focus on inflammatory suppression but frequently lack efficacy in driving mucosal healing. Moreover, prolonged corticosteroid use is associated with significant risks, including increased susceptibility to infections, steroid dependence, and osteoporosis. Conversely, dental pulp stem cells (DPSCs) have dual therapeutic capabilities: precise immune modulation and accelerated tissue regeneration.

**Figure 1:**
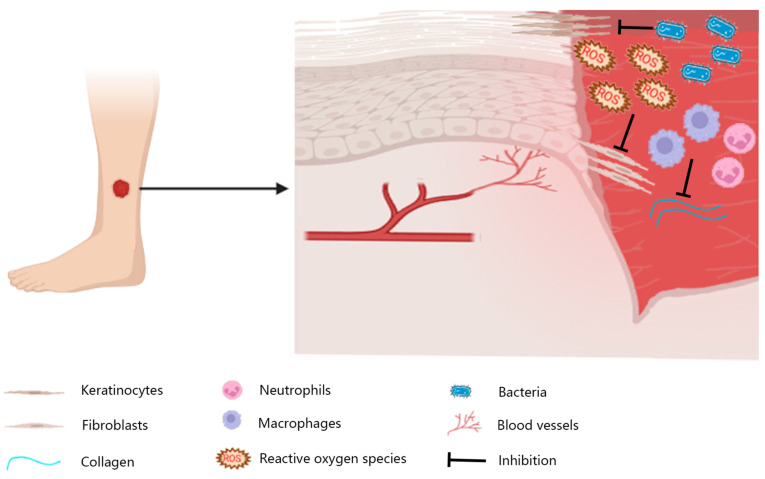
Wounds on the skin of the lower extremities are usually chronic. Local inflammation, bacterial infections, and reactive oxygen species can lead to impaired vascularization, insufficient granulation tissue production, and re-epithelialization stagnation.

Wound healing is a complex physiological process. It involves the temporal and spatial synchronization of various cells that perform different functions at different times [7]. The stages occur in chronological order but also overlap and influence each other [8]. In addition to scarring, chronic wounds are also common in wound healing. Chronic wounds are those in which the repair process fails to restore anatomic and functional integrity after three months [9]. Prolonged inflammation is an important factor contributing to the difficulty of wound healing (Figure 1). During the inflammatory phase of acute wound healing, M1 macrophages (pro-inflammatory macrophages) predominate. To facilitate accelerated healing, M1 macrophages gradually transition to M2 macrophages (anti-inflammatory macrophages). M2 macrophages promote wound healing by stimulating angiogenesis, facilitating extracellular matrix (ECM) deposition, and modulating the inflammatory microenvironment [7]. Macrophages transform from M1 to M2 after engulfing activated neutrophils. However, in chronic wounds, 80% of M1 macrophages that are unable to phagocytose spent neutrophils are observed [10]. Neutrophils contain large amounts of ECM-degrading enzymes, including elastase and matrix metalloproteinases (MMPs) [11]. Impaired re-epithelialization is also a factor in prolonging wound healing, and keratinocytes (KCs) play a key role in re-epithelialization. Overexpression of miRNA-21, miRNA-132, and miRNA-31 can promote KCs’ proliferation and migration [12]. Disorders of blood vessel formation are also factors that delay wound healing. Angiogenesis involves cytokines such as angiotensin, fibroblast growth factor, vascular endothelial growth factor (VEGF), hypoxia-inducible factor-1α (HIF-1α), and stromal cell-derived factor-1 [13]. The common pathogens in chronic wounds are predominantly Staphylococcus aureus and Pseudomonas aeruginosa [14]. This leads to prolonged inflammation of the wound and the release of large amounts of reactive oxygen species (ROS), resulting in delayed wound healing. ROS stimulate sustained TNF-α production, thereby inducing chronic inflammation. Furthermore, ROS upregulate MMP expression in dermal fibroblasts via the activation of Nuclear Factor-κB (NF-κB) and activator protein 1 (AP-1), a process that contributes to delayed wound healing [15]. In addition, systemic factors such as age [16], stress [17], diabetes [18], medication, obesity [19], alcohol consumption [20], smoking [21], and nutrition [22] affect wound healing.

The purpose of this review is to elucidate the mechanism by which DPSCs accelerate the healing of skin and mucosal wounds and evaluate strategies for combining DPSCs with biomaterials to enhance therapeutic efficacy, providing a basis for the development of new methods for promoting healing based on DPSCs and serving clinical needs.

## 2. Dental Pulp Stem Cells and Derivatives

DPSCs are mesenchymal stem cells with high proliferative capacity, low immunogenicity, and multidirectional differentiation properties. DPSCs are routinely isolated from the pulp tissues of impacted third molars and orthodontically extracted premolars. DPSCs express mesenchymal stem cell-specific markers (CD105, CD90, and STRO-1) [23]. DPSCs have the potential to regenerate blood vessels [24]. Unlike acellular derivatives, DPSCs incur substantial expenses for cryopreservation in liquid nitrogen (−196 °C) and carry potential tumorigenic liabilities. DPSC-conditioned medium (DPSCs-CM) contains many cytokines, chemokines, and growth factors. Compared with Bone mesenchymal stem cells (BMSCs)-CM, DPSCs-CM contained higher levels of Interleukin-10 (IL-10), IL-13, and transforming growth factor-β1 (TGF-β1). However, hepatocyte growth factor, adiponectin, MMP-13, neurotrophic factor-3, and MMP-9 were specific to DPSC-CM. DPSC-CM has therapeutic potential for diseases affecting multiple organs and tissues, including the heart, lung, liver, eye, bone, cartilage, nerve tissues, and dental tissues [25]. DPSCs-Extracellular Vesicles (DPSCs-EVs) represent a nanoscale extracellular vesicle subtype (40–150 nm) that mediates cell-cell signaling through paracrine shuttling of bioactive payloads, including mRNAs/miRNAs/proteins, between cellular populations [26]. DPSCs-EVs regulate inflammation, promote tissue repair and regeneration, and can be used as drug carriers. Since DPSCs-EV yields are low and difficult to obtain, the lysate compensates for these shortcomings. Several studies have reported that DPSC lysates are effective in treating colitis [27] and diabetic chronic wounds [28]. Artificial Cell-Derived Vesicles (ACDVs) extracted from DPSCs lysates are also nanoscale particles. Compared with DPSCs-EVs extracted by ultrafast centrifugation, ACDVs exhibited 16 times higher extraction efficiency. It shares a similar protein secretion profile with DPSCs-EVs [29].

## 3. Mechanisms

**Figure 2:**
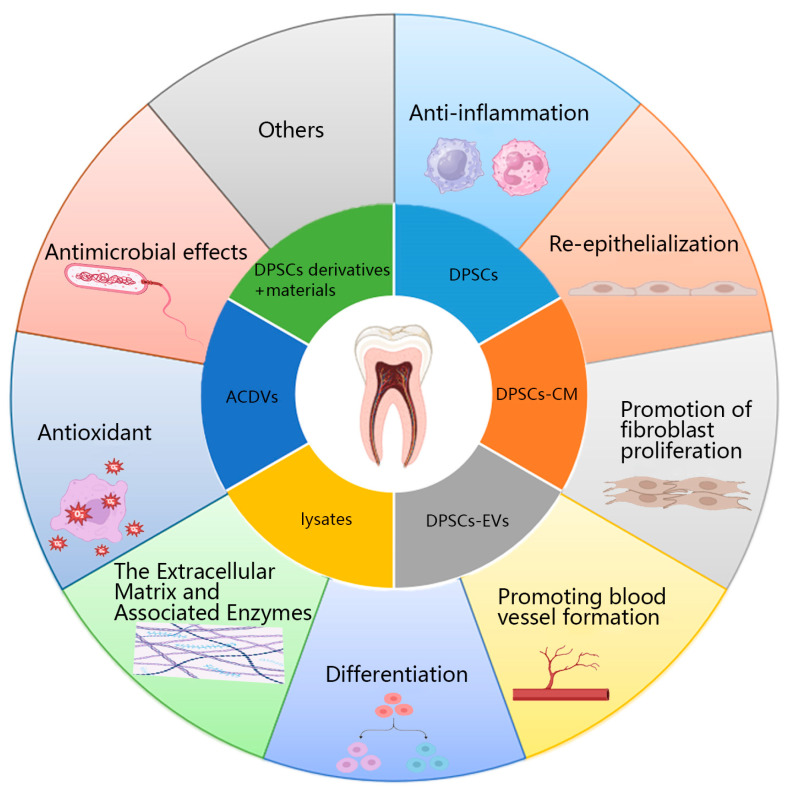
Mechanism of action of DPSCs and their derivatives in promoting wound healing. DPSCs: Dental pulp stem cells; DPSCs-CM: DPSCs-conditioned medium; DPSCs-EVs: DPSCs-extracellular vesicles; ACDVs: Artificial cell-derived vesicles.

DPSCs and their derivatives accelerate skin wound healing through various mechanisms (Figure 2).

### 3.1. Anti-Inflammation

While inflammatory responses are essential for eliminating pathogens and necrotic tissue, excessive or prolonged inflammation can hinder healing. Clinical debridement, which establishes a fresh wound bed, is thus beneficial for promoting healing.

DPSCs and their derivatives can improve the inflammatory environment of wounds and accelerate healing. Greene et al. applied DPSC products to a wound in vivo model of diabetc mice. They observed that DPSCs products reduced the nuclear transfer of NF-κB, promoted the phosphorylation of SMAD family member 2 (SMAD2) and SMAD3, upregulated IL-10 but reduced IL-6 expression, and significantly promoted wound healing [30]. Duan et al. demonstrated that ACDVs treatment (20 μg/mL) downregulated IL-1β and upregulated IL-10, exhibiting a mechanism similar to that of DPSC-EVs (20 μg/mL) in both in vivo and in vitro wound models, with enhanced healing kinetics [29]. Yang et al. showed that DPSCs have greater tissue regeneration potential than periodontal ligament stem cells, adipose-derived stem cells (ADSCs), and BMSCs, and that DPSCs and DPSC-CM can promote macrophage M2 polarization. DPSC-secreted chemokine ligand 2 (CCL2) drives M2 macrophage polarization while enhancing mucosal repair through coordinated healing mechanisms in vivo [31]. Anderson et al. stimulated DPSCs with pro-inflammatory substances for 4 h, and their expression of anti-inflammatory-related factors was enhanced. In simulated in vitro wounds of intestinal epithelial cells, DPSC and stimulated DPSC accelerated healing by upregulating the extracellular signal-regulated kinase 1/2 (ERK1/2) pathway. When co-cultured with macrophages, DPSCs secrete kynurenine. Kynurenine is transported into macrophages through the L-type amino acid transporter 1, where it stimulates PPARγ expression, thereby inducing the expression of genes associated with M2 polarization [32]. Under inflammatory conditions, DPSC-CM increases the proportion of M2 macrophages [33]. Microglia are macrophages that reside in the nervous system. DPSCs can downregulate tumor necrosis factor-α (TNF-α), cyclooxygenase-2, IL-1β, MMP9, and IL-6, and upregulate IL-10 and Arg in microglia. DPSCs inhibit cellular energy metabolism and proliferation by blocking cell mitosis [34]. In a separate study, Duan et al. demonstrated that lysates from zeolitic imidazolate framework-8 (ZIF-8)-loaded DPSCs suppressed continuous ultraviolet irradiation-induced IL-1β expression and neutrophil infiltration [35].

DPSCs and their derivatives have been shown to possess significant anti-inflammatory properties, which are the key mechanisms behind their ability to accelerate wound healing in both skin and mucosal tissues. Furthermore, their anti-inflammatory action extends to the nervous system, where they can attenuate neuroinflammation mediated by the activation of microglia. Within the diabetic corneal epithelium, neurotrophic factors such as substance P, vasoactive intestinal peptide, and related neurogenic mediators secreted by nerve cells can accelerate epithelial healing and regulate inflammation [36]. This synergistic effect is beneficial for the repair of epithelial tissues.

### 3.2. Re-Epithelialization

Macrophages secrete IL-1 and TNF-α, promoting dermal fibroblasts to secrete keratinocyte growth factors (KGF) and IL-6. This process causes KCs to become flat and elongated, forming peripheral actin filaments that facilitate their migration to the wound site [37].

Xu et al. created a hydrogel with DPSCs lysate that promoted the proliferation, migration, and keratinization of KCs, thereby promoting the regeneration of the epidermal layer of diabetic wounds [28]. Gelatin methacryloyl (GelMA) hydrogels containing DPSCs and VEGF can also accelerate wound re-epithelialization and promote wound healing [38]. Greene et al. observed significant expression of collagen and fibronectin and fibroblast recruitment in wounds of diabetic mice treated with DPSCs products, and wound re-epithelialization was significantly earlier than that in controls [30]. Martínez-Sarrà et al. treated the back wound of each athymic nude mouse with 1 × 10^6^ DPSCs or Phosphate Buffered Saline (PBS), and the epithelium was completely recovered and close to the thickness of normal skin in the DPSCs-treated mice, while only 40% of the wound epithelium in the PBS-treated [39].

Early-stage inflammation facilitates keratinocyte mobilization, alongside the debridement of necrotic debris and pathogens. DPSCs demonstrate dual regenerative capacities by enhancing keratinocyte migratory dynamics while restoring epidermal barrier homeostasis. Notably, the synergistic delivery of hydrogel-VEGF composites potentiated epithelialization kinetics through biomimetic niche engineering. Biomimetic niches are microenvironments that promote cell survival and function. Their role is analogous to that of the ECM. VEGF promotes keratinocyte metabolism during wound healing by providing oxygen and nutrients to the cells. The GelMA hydrogel accelerates ECM deposition and re-epithelialization by enhancing cell migration, proliferation, and adhesion and providing a supportive framework.

### 3.3. Promotion of Fibroblast Proliferation

Fibroblasts and other components constitute granulation tissue, which provides the basis for re-epithelialization. When stimulated by TGF-β, they differentiate into myofibroblasts, which effectively promote wound healing through the contraction of myofibroblasts. Both myofibroblasts and fibroblasts secrete ECM, providing a reticular framework for cell growth, storing growth factors, and transmitting mechanical and biological signals [40].

DPSCs promote fibroblast proliferation and collagen synthesis by secreting TGF-β [41]. DPSCs exhibit stronger biological effects after drug stimulation. Chin et al. demonstrated that 2,3,5,4′-tetrahydroxystilbene-2-O-β-D-glucoside enhances the proliferative influence of DPSC-CM on human gingival and skin fibroblasts. It reduces the release of pro-inflammatory factors stimulated by lipopolysaccharides (LPS). This regimen significantly improved wound healing [42].

Granulation tissue functions as a dynamic biosystem that orchestrates cellular dynamics and molecular trafficking. DPSC-derived TGF-β secretion potently stimulates fibroblast proliferation while facilitating the architectural development of this provisional matrix, ultimately establishing functional regenerative scaffolds.

### 3.4. Promoting Blood Vessel Formation

Maintaining vascular stability and maturation is important for the physiological function of blood vessels.

DPSCs promote endothelial cell (ECs) migration and angiogenesis by promoting VEGF expression. Conversely, TGF-β1-treated DPSCs reduce VEGF expression, inhibit ECs migration, and enhance vascular stability [43]. Consequently, DPSCs critically upregulated the expression of angiogenic factors. Zhou et al. demonstrated that DPSC-EVs (10 μg/mL) activate the cell division cycle 42/p38 mitogen-activated protein kinase (Cdc42/p38 MAPK) cascade via VEGF-VEGFR2 binding, enhancing human umbilical vein endothelial cell (HUVEC) migration and stimulating neovascularization. This conclusion was further confirmed by the in vivo application of a p38 inhibitor [44]. Binding to VEGF, VEGFR2 phosphorylates Y1214, sequentially activates downstream Cdc42 and p38, triggers actin aggregation, integrates into stressed fibers in ECs exposed to VEGF, causes EC migration, and promotes germination of blood vessels [45]. DPSCs can also promote blood vessel formation in response to changes in their external environment. Li et al. found that hypoxia-treated DPSC-EVs promote angiogenesis and upregulate lysyl oxidase 2, which may be related to the regulation of VEGFA, MMP2, MMP9, and stromal cell-derived factor-1α angiogenesis factor in HUVECs. In contrast, silencing lysyl oxidase 2 reduces the angiogenic effects of hypoxia-treated DPSC-EVs (10 μg/mL) in vitro [46]. DPSCs-EVs (100 μg/mL) damaged by periodontitis can promote ECs proliferation, migration, and angiogenesis more strongly than healthy DPSCs-EVs [47].

DPSCs activate the p38 MAPK pathway via VEGF-VEGFR signaling to promote angiogenesis. Hypoxic and periodontitis microenvironments augment DPSCs’ angiogenic capacity.

### 3.5. Differentiation

DPSCs are multipotent adult stem cells with the potential to differentiate into adipogenic, osteogenic, and chondrogenic lineages [35].

Zhang et al. cultured DPSCs in a filtered HUVEC-conditioned medium and observed significant upregulation of von Willebrand factor (vWF), CD31, and VEGFR2 compared to DPSCs cultured alone. TGF-β1 induces DPSCs to differentiate into pericyte-like cells, enhancing vascular stability and promoting wound healing [43]. For spinal cord injury, ZIF-8/DPSCs/GelMA composite hydrogels enhanced DPSCs’ neural differentiation potential and vascular regeneration through c-Jun N-terminal Kinase 1 (JNK1) and p38 MAPK signaling, demonstrating greater neuroprotective efficacy than Zn^2+^ alone [48]. Almost all wounds are accompanied by damage to the skin appendages. Once hair follicles are damaged, hair loss becomes irreversible. While DPSC-CM has demonstrated the ability to delay chemotherapy-induced alopecia and promote hair regrowth [49], its effect on true follicular regeneration remains unsubstantiated, warranting further investigation.

The MAPK signaling pathway governs three cardinal cellular processes: proliferation, differentiation, and apoptosis. DPSCs can differentiate into pericyte-like cells to enhance vascular stability. DPSC can also promote neural differentiation and angiogenesis through the JNK1 and p38 MAPK signaling pathways. Although limited by the availability and viability of hair follicles, hair transplantation is an effective therapy for treating hair loss [50]. The role of DPSCs in hair follicle regeneration remains unexplored in the existing literature. Given the substantial clinical demand for hair follicle regeneration, an in-depth investigation of DPSCs’ potential applications in this field is imperative.

### 3.6. The Extracellular Matrix and Associated Enzymes

DPSCs induce the polarization of M2 macrophages, which can not only inhibit inflammation and prevent the formation of chronic wounds but also accelerate ECM deposition and promote wound healing by promoting collagen production. During wound healing, MMP and tissue inhibitor of metalloproteinase-1 (TIMP-1) are involved in many processes, such as vascular germination, ECM contraction, fibroblast and KCs migration, and re-epithelialization.

Topical application of DPSC-derived products in diabetic murine wound models activates fibroblast SMAD signaling, upregulating α-smooth muscle actin (α-SMA), fibronectin, and collagen I (COL I) expression while enhancing ECM deposition [30]. Zanini et al. stimulated fibroblasts with TNF-α, IL-1β, or LPS. Co-culturing with DPSCs regulated the levels of fibrosis-associated genes (TGF-β, fibronectin 1, COL I, and COL III) and proteins associated with ECM deposition and remodeling (α-SMA, fibronectin, and MMP-9). Given that the anti-inflammatory effect of dexamethasone is determined, the stimulation of the collagen gene by DPSCs is influenced by inflammation [51]. In diabetic wounds, elevated IL-1β sustains chronic inflammation while suppressing fibroblast activity, which is a critical process that stalls tissue repair. Through p38 MAPK signaling, this cytokine disrupts ECM homeostasis by upregulating MMP and downregulating TIMP. Concurrently, IL-1β impairs collagen I/III synthesis, compromising ECM structural integrity and creating a self-perpetuating cycle of impaired wound healing [52]. Wu et al. found that overexpression of sarcoma proto-oncogene (Src) promotes KCs migration by increasing MMP-2 and decreasing E-cadherin expression through the ERK1/2 pathway. Wound healing is slowed down in vivo and in vitro by silencing Src [53]. In the skeletal system, MMP-9 acts as an osteoclast marker, and DPSCs can reduce its expression [54]. DPSC-CM can stimulate the expression of TIMP-1 in mouse articular chondrocytes [55]. Macrophages are crucial for the formation of ECM. It has both pro-fibrotic and anti-fibrotic effects on fibroblasts. Anti-fibrosis is mainly achieved through the secretion of MMP-related proteolytic enzymes and phagocytosis of ECM components [56]. M1 macrophages activate fibroblasts by secreting pro-inflammatory factors, thus promoting the production of MMP. M2 macrophages secrete TGF-β1 to promote the transcription of α-SMA in fibroblasts and promote their transformation into myofibroblasts and ECM remodeling. M2 macrophages can also activate glutamate and proline by the production of arginase, thus promoting collagen synthesis [57]. Prolonged exposure to ultraviolet rays can lead to the loss of collagen. ZIF-8 encapsulated DPSC lysates can significantly reduce the loss of collagen. Under the function of ZIF-8, the DPSCs lysate is more stable and long-lasting [35].

DPSCs mediate ECM remodeling through MMP-TIMP regulatory mechanisms, a process intrinsically linked to the inflammatory cytokine networks and fibroblast interactions.

### 3.7. Antioxidant

Oxidative stress is determined by the body’s ROS and levels of antioxidants. The main source of ROS is the oxidative respiratory chain in the mitochondria. During hemostasis, appropriate ROS levels promote the adhesion and aggregation of platelets, thereby promoting platelet activation [58]. At physiological levels, ROS enhance angiogenesis, epithelial repair, and neutrophil recruitment. ROS released by neutrophils and macrophages destroy bacteria to prevent infection. However, excessive ROS delays wound healing by inhibiting the effects of cytokines, such as VEGF and TNF-α [59].

Deng et al. delineated the pathological impact of oxidative stress on diabetic complications (cutaneous damage, neuropathy, peripheral ischemia, and localized infections) and characterized the therapeutic potential of antioxidants in enhancing diabetic wound repair [60]. Guan et al. designed a continuous oxygenation system capable of delivering oxygen and scavenging ROS. The system promoted the survival and migration of KCs and fibroblasts under hypoxic conditions. ECs in diabetic wounds can survive and form capillaries while reducing the infiltration of inflammatory cells in the wound area [61]. Mishra et al. found that DPSC-derived mitochondria act as neuroprotective agents. In a model of Alzheimer’s disease, it was able to reduce the ROS production of amyloid beta peptide and streptozotocin [62].

Hence, we speculate that the role of DPSCs in ROS clearance may also affect wound healing.

### 3.8. Antimicrobial Effects

The effect of skin microbes on wound healing is bidirectional. Group A streptococcus accelerates KCs’ chemotaxis and wound re-epithelialization by activating plasminogen. Pseudomonas colonizes epithelial tissues to increase angiogenesis and accelerate epithelialization via KGF-1. Under infectious conditions, Pseudomonas aeruginosa colonizing the skin induces cell apoptosis via the TAK1/MKK/p38 signaling pathway, impairing tissue repair. In the absence of infection, this bacterium suppresses apoptosis and enhances wound healing. Corynebacterium protects the epidermis from ROS damage by producing superoxide dismutase [63].

Canesso et al. reconstructed the intestinal microbiota of mice and found that the wounds of germ-free mice healed faster and without scars [64]. Afami et al. found that peptide hydrogels loaded with DPSCs exerted antibacterial activity by breaking the biofilms of common oral-colonizing bacteria, such as Staphylococcus aureus, Enterococcus faecalis, and Fusobacterium nucleatum [65].

While current research scarcely addresses microbial modulation in cutaneous wound healing through DPSC-based interventions, emerging evidence highlights the therapeutic potential of these stem cell populations in microbiome-targeted regenerative applications.

### 3.9. Others

Local mechanical forces cause scarring at the end of wound healing. Can DPSCs reduce scar width by adjusting mechanical stimulation at a later stage? Under the stimulation of local tension, the upregulation of TGF-β promotes the differentiation of DPSCs into fibroblasts, which enhances the resistance of the local skin to stretching. Mechanical force can promote the proliferation of DPSCs, and appropriate mechanical stimulation can activate antioxidant defense enzymes [66]. While ROS impair wound repair processes, the mechanical preconditioning of DPSCs demonstrates therapeutic potential through antioxidant enzyme activation. Further research is needed to determine whether it can alter fibroblast and macrophage types by resisting mechanical forces. When local tension stimulates mechanical receptors on the cell membrane, the upregulation of TGF-β promotes fibroblast differentiation into myofibroblasts by activating adhesion point kinases. They secrete a large amount of ECM, mainly composed of COL I. Simultaneously, inhibiting the action of MMP leads to excessive deposition of ECM. Eventually, there was an increase in the scar area. Clinically, tension-relieving strips are applied during the remodeling phase to mitigate this process. In addition, mechanical force can increase the scar area by activating the YAP gene [67]. After knocking down EN-1, hair follicles and glands can be observed in EN-1-negative fibroblasts [68]. M2 macrophages can promote wound healing during the proliferative phase and ECM deposition. Actin and myosin contract in the ECM, elongate macrophages, and induce M2 macrophage polarization [69]. This can further accelerate wound healing.

Hypertension, a prevalent multisystem disorder, induces multiorgan pathology, notably impacting the cardiovascular, neurological, and renal systems. Hypertension damages endothelial cells and triggers vascular inflammation, ultimately leading to arteriolosclerosis [70]. This often negatively impacts wound healing, causing Martorell hypertensive ischaemic leg ulcers [71]. In addition to their primary vasodilatory effects, current antihypertensive medications work through mechanisms such as reducing inflammation, modulating MMP activity, and combating ROS [72]. DPSCs also exhibit similar therapeutic properties. Studies have shown that hypertension may aggravate scar hyperplasia and keloid formation [73]. At present, there is a lack of direct evidence that DPSCs improve high blood pressure. However, DPSCs can repair ECs, promote nitric oxide release, and improve heart disease. When blood fills the cavities of the cavernous body, it enables normal penile erection [74]. Hypertension destroys ECs, and the production of nitric oxide decreases, so that the blood vessels of the cavernous body cannot dilate, resulting in erectile dysfunction. DPSCs-CM can repair EC damage and improve erectile function [75]. The compensatory phase of hypertension can lead to ventricular hypertrophy, which can cause myocardial damage over time. Cardiomyocytes are permanent cells that can only be replaced by fibroblasts after injury, eventually leading to scar formation. DPSCs-EVs (2000 μg/mL) can induce DPSCs to differentiate into cardiomyocytes, improve various cardiomyocyte injuries [76], and prevent scar repair. Hypertension damages cardiomyocytes, causing them to thicken and reducing the cardiac output. This activates the renin-angiotensin system, worsening the hypertension. The potential of DPSC differentiation into cardiomyocytes can break this vicious cycle and indirectly improve hypertension. Based on the previous introduction that hypertension inhibits wound healing, is the mechanism by which DPSC accelerates wound healing related to its regulation of hypertension?

A 10–60 mV transepidermal potential gradient, actively maintained by epidermal Na^+^/K^+^-ATPase activity, exists between the dermal and epidermal layers. When the skin is damaged, an endogenous current is generated that plays a key role in wound healing. In vitro, electrical stimulation has been observed to exert an antibacterial effect, promote the migration of macrophages, fibroblasts, and neutrophils, improve blood perfusion, and promote angiogenesis and wound re-epithelialization. It also plays a role in chronic wound healing [77]. Under maximum output current (12.6 μA), a conductive hydrogel with DPSCs exhibits three key improvements over non-stimulated controls: (1) enhanced stem cell characteristics (elevated OCT-4/SOX-9 mRNA); (2) promoted angiogenesis (increased VEGFR/VEGFA); and (3) Induced M2 polarization (higher CD206 via immunofluorescence/flow cytometry) [78]. Electrical stimulation opens a whole new path for wound healing.

Organoids are stem cell derivatives that are 3D-structured multicellular tissues produced from primary tissues or stem cells. It can simulate the organization and function of actual organs. A variety of mature models can be built [79]. DPSCs can generate organoids, including cortical organoids [80], midbrain-like organoids [81], and dental pulp organoids [82]. These findings provide more options for modeling or repairing neurological and oral diseases. For the skin and mucous membranes, if it is difficult to establish a complete structure, a layered structure can be considered. Organoids are a promising treatment approach that enhances tissue wound healing through tissue engineering and regeneration.

## 4. Scaffold Materials in Wound Healing

The combination of DPSCs with bioengineering materials, representing a derivative approach, leverages the mechanisms described above. Building upon this foundation, Table 1 summarizes the applications of DPSC-material constructs in wound healing.

## 5. DPSCs and Derivatives for Mucosal Repair

Compared with skin tissue, the oral mucosa can heal quickly and form fewer scars, which is also related to saliva. Saliva is an indispensable physiological medium in oral tissue regeneration. Saliva is an almost neutral buffer with a pH value between 6 and 7.5, which can act as a lubricant, similar to wet healing of wounds. The antimicrobial peptides in saliva can inhibit the growth of various microorganisms, promote cell migration, regulate immune function, and accelerate wound healing [89]. Leptin in saliva promotes the proliferation [90] of keratinocytes. Resident microorganisms in the oral cavity are beneficial for wound healing, as saliva provides nutrients, accelerates the healing of oral mucosal wounds, and results in smaller scars in the later stages. The hard palate mucosa exhibits excellent healing ability. Compared to skin microbiota, oral bacteria enhance mesenchymal stem cell migration and proliferation while reducing inflammation. In addition, pro-repair mechanisms involve regulated autophagy, enhanced ECM deposition, and induced macrophage polarization [91].

### 5.1. Oral Mucosa

DPSCs can secrete CCL2 to promote M2 polarization, and even senescent DPSCs at the 8th passage retained the ability to secrete CCL2 to induce M2 polarization. DPSC-CM alone also promoted M2 polarization. At equivalent CM concentrations, DPSCs-CM exhibited significantly greater M2 macrophage enrichment than BMSCs-CM. In this study, injecting DPSCs into the mucosal tissue at the gingival margin accelerated mucosal repair, which could be inhibited by a CCL2 neutralizing antibody [31]. This indicates that during wound healing, both skin and mucosal M2 macrophages effectively accelerate tissue repair. Currently, there is a large population of betel nut chewers, and arecoline in betel nuts damages the oral mucosa and increases the risk of oral cancer. DPSC-CM significantly inhibited arecoline-induced apoptosis of oral mucosal epithelial cells. However, in oral cancer models fabricated with 4-nitroquinoline-1-oxide, DPSCs may drive the malignant transformation of oral precancerous lesions by activating the mitochondrial mTOR signaling pathway. Consequently, DPSC-based cell therapy should be avoided in oral precancerous lesions [92]. In a scratch test using an in vitro wound healing model of gingival fibroblasts, the DPSCs secretion protein group transfected with TGF-β1 significantly promoted the migration of gingival fibroblasts, which was more pronounced and statistically significant than that in the control secretion group [93]. Nickel is a commonly used metal in dentistry for the reconstruction of dental function. Peri-implant inflammation [94] and hypersensitivity reactions [95] are the most common adverse effects associated with the placement of foreign bodies in the oral cavity. By establishing a nickel hypersensitivity model in the oral cavity, the loading of DPSCs-EVs with catechol-chitosan hydrogel can effectively increase the Th2/Th1 ratio and control the hypersensitivity reaction of nickel in the oral cavity. The DPSCs-EV hydrogel group significantly reduced the inflammatory response of the buccal mucosa [96], which is conducive to mucosal tissue repair.

### 5.2. Esophageal Mucosa

According to the data from the China National Cancer Center, there were 224,000 cases of esophageal cancer in 2022, with a crude incidence rate of 15.87 per 100,000, and 187,500 deaths from esophageal cancer in the same year [97]. Radiation therapy is indispensable for treating esophageal cancer [98]. However, radiation therapy can damage the mucosa of the esophagus. To address this potential complication, an animal model of esophageal radiation injury was used. In this model, intravenous DPSC administration attenuated mucosal pro-inflammatory cytokines (IL-1β, TNF-α, IL-8) while inducing marked mucosal thickening, demonstrating dual anti-inflammatory and tissue-restorative capacities. DPSCs significantly improved injury and recovery of the esophageal mucosa, which was also reflected in an increase in the rats’ daily food intake [99].

### 5.3. Colonic Mucosa

Mesenchymal stem cells (MSCs) constitute a key investigative focus in UC therapeutics [100], with ADSCs [101], umbilical cord stem cells [102], intestinal stem cells [103,104,105], embryonic stem cells [106], and human amniotic stem cells [107] demonstrating significant therapeutic potential through diverse delivery methods. Hepatocyte growth factor has been shown to effectively promote intestinal mucosal repair [107]. DPSCs overexpressing hepatocyte growth factor can transdifferentiate into intestinal epithelial-like cells, promote their proliferation, suppress inflammatory cytokines (TNF-α, interferon-γ), and enhance the expression of the anti-inflammatory cytokine IL-10 while reducing oxidative stress damage to facilitate UC repair [108]. In a colitis model induced by dextran sodium sulfate, DPSCs induced T cell apoptosis by expressing Fas ligands, reducing colon inflammatory cell infiltration, and secreting inflammatory cytokines. This result was also confirmed by the knockdown of the Fas ligand in DPSCs [109]. As a representative para-aminosalicylic acid drug, sulfasalazine is the first-line clinical treatment for mild UC. In experimental studies by Aly et al., the combination of sulfasalazine and DPSCs alleviated colonic inflammation and accelerated mucosal repair by modulating the inflammatory microenvironment and reducing reactive oxygen species levels, with effects more pronounced than treatment alone [110].

### 5.4. Fallopian Tubes

The fallopian tubes serve as a channel for ovum transport and fertilized egg implantation, and infertility of the fallopian tubes due to tubal infection [111], surgery [112], and the epidemic of sexually transmitted diseases [113] has shown an upward trend. Ovulation induction drugs may be considered for ovulatory dysfunction [114], but structural abnormalities must be surgically treated. The recurrence rate is high two years after surgery [115], and better treatment is needed for this condition. Thermosensitive Pluronic F-127 forms a porous [116] and degradable hydrogel [117] in the human body with good cytocompatibility [117] and is widely used in bioengineering research [118]. Luo et al. were modeled by injecting absolute ethanol into the fallopian tubes, and the DPSCs were encapsulated with Pluronic F-127 hydrogel. DPSC-containing hydrogels can promote vascular endothelial growth factor secretion, accelerate angiogenesis, regulate inflammation, and inhibit apoptosis, thereby accelerating the repair and regeneration of the fallopian tube mucosa [119].

## 6. Prospect

DPSCs are easily obtained from sources such as deciduous teeth and third molars. As mentioned earlier, DPSCs and their derivatives have shown great potential for skin and mucosal healing. When combined with other materials or drugs, they deliver even better results. Examples include ZIF-8, GelMA, sulfasalazine, and Pluronic F-127 hydrogels.

The mucosa, which lines the cavity organs, is crucial for protection and piping. Unlike skin damage, mucosal damage rarely affects appearance but causes functional problems. For instance, long-term oral mucosal damage may lead to oral cancer, esophageal and intestinal mucosal damage can hinder food intake and absorption, and fallopian tube damage can cause infertility.

Numerous studies have investigated the role of DPSCs in skin wound healing, and certain mechanisms have already been elucidated. Further exploration of their accelerated healing mechanisms could not only advance their application in mucosal tissue repair but also suggest novel therapeutic approaches. In wound repair, closing wounds is the first step, but scarless healing is the real goal. Saliva may aid in oral mucosa repair without scarring. Oral epithelial cells have low TGF-β1 levels; therefore, oral mucosal scars are rarer than skin scars [120]. In addition, fetal skin damage in early pregnancy can fully regenerate without scarring [121]. However, adult wounds scar more, possibly because TGF-β1 and TGF-β2 dominate and TGF-β3 is low [11]. Fetal wounds have a higher type III/type I collagen ratio and more hyaluronic acid [122]. In a clinical trial involving patients with acne, those who developed scars exhibited significantly heightened inflammatory responses and markedly elevated TGF-β1 levels compared to patients without scarring [123]. A separate double-blind clinical trial demonstrated TGF-β3’s impact on scarring. Patients undergoing saphenofemoral junction ligation and great saphenous vein stripping for varicose veins received intradermal injections of TGF-β3 in one thigh and a placebo in the contralateral thigh on the day of surgery. When assessed by researchers, patients, and independent observers at 6 weeks to 7 months postoperatively, 500ng TGF-β3 significantly improved scar appearance compared to placebo [124]. Modulating inflammatory responses, inducing M2 macrophage polarization, coordinating proliferative regulation, and optimizing the TGF-β3/β1 and collagen III/I ratios enable scarless tissue regeneration. After evaluating various treatments for safety, efficacy, and cost, our goal is to apply these findings in clinical practice. This will improve mucosal injury treatment and benefit patients by promoting scarless skin and wound healing.

## 7. Conclusions

Analysis of the mechanisms by which DPSCs function in the skin and mucosal tissues reveals similarities in their ability to accelerate tissue repair. Furthermore, clinical studies suggest that by modulating TGF-β signaling and inflammatory responses, DPSCs hold promising potential for achieving near-scarless healing.

## Figures and Tables

**Table 1:** Combination of scaffold materials and DPSCs in wound healing.

Authors	Year	Combination Preparation	Results
Williams et al. [83]	2013	HEMA	Scratch and Transwell assays showed that HEMA (<3 mM) dose-dependently suppressed DPSCs migration, which impaired pulp healing.
Khayat et al. [84]	2017	GelMA wraps DPSCs and HUVECs	The binding of GelMA hydrogel to DPSCs/HUVECs favors the formation of neovascularity.
Badhe et al. [85]	2021	Conducting-Polymer-Based Hydrogel Dressing loaded with DPSCs	The hydrogel provides a moist environment, a 3D matrix for cells to migrate freely, and the antimicrobial activity of chitosan. These factors contribute to electrotherapy to accelerate wound healing in vitro and in vivo.
Matheus et al. [38]	2023	GelMA equipped with DPSCs and VEGF	GelMA loaded with DPSCs and VEGF could promote wound healing, and increased gene expression of Keratin 10 within 4 weeks after healing.
Dasgupta et al. [86]	2023	Combination of chitosan-collagen-fibrinogen and vitamin D3	Vitamin D3 binds to chitosan, collagen, and fibrinogen and crosslinks with ultraviolet light to induce DPSCs to differentiate into ECs via the HIF-1/IGF-1/VEGF pathway.
Lu et al. [87]	2024	GelMA hydrogel combined with DPSCs and FGF21	It promotes the transformation of N-adhesion protein to E-adhesion protein and accelerates epithelial formation by recruiting epidermal adhesion protein; promotes angiogenesis and increases wound blood perfusion; regulates lysosomal stability, activates autophagy, maintains intracellular homeostasis, and comprehensively encourages the recovery of diabetic burns.
Davydova et al. [88]	2024	Polysaccharide complex alginate-pectin hydrogel	Not only did the alginate-pectin polysaccharide hydrogel maintain moist conditions promoting KCs and fibroblast proliferation, but it also did not compromise DPSCs’ proliferative capacity.

**Abbreviations:** GelMA: Gelatin methacryloyl; IGF-1: Insulin-like growth factor-1; DPSCs: Dental pulp stem cells; FGF21: Fibroblast growth factor 21; HEMA: 2-Hydroxyethyl methacrylate; HIF-1, hypoxia-inducible factor-1; HUVECs: Human umbilical vein endothelial cells; VEGF: Vascular endothelial growth factor; ECs, endothelial cells; KCs: Keratinocytes.

## Abbreviations

The following abbreviations are used in this manuscript:

ACDVs	Artificial Cell-Derived Vesicles
ADSCs	Adipose-derived stem cells
Arg	Arguments
BMSCs	Bone mesenchymal stem cells
CM	Conditioned Medium
CCL2	Chemokine ligand 2
COL I	Collagen I
DPSCs	Dental Pulp Stem Cells
ECs	Endothelial cells
ECM	Extracellular matrix
EVs	Extracellular Vesicles
FGF	Fibroblast growth factor
GelMA	Gelatin methacryloyl
HEMA	2-Hydroxyethyl methacrylate
HIF-1	Hypoxia-inducible factor-1
HUVECs	Human umbilical vein endothelial cells
IGF-1	Insulin-like growth factor-1
IL-10	Interleukin-10 (IL-10)
KCs	Keratinocytes
KGF	Keratinocyte growth factors
LPS	Lipopolysaccharide
M2	M2 polarization
MMP	Matrix metalloproteinase
miR	microRNA
MAPK	Mitogen-Activated Protein Kinase
NF-κB	Nuclear Factor kappa-B
PBS	Phosphate Buffered Saline
PPARγ	Peroxisome proliferator-activated receptor γ
ROS	Reactive oxygen species
TGF-β	Transforming Growth Factor-β
TIMP	Tissue Inhibitor of Metalloproteinase
TNF-α	Tumor Necrosis Factor-α
UC	Ulcerative Colitis
VEGF	Vascular Endothelial Growth Factor
ZIF-8	Zeolitic imidazolate framework-8

## References

[1] GBD 2021 Diabetes Collaborators (2023). Global, regional, and national burden of diabetes from 1990 to 2021, with projections of prevalence to 2050: A systematic analysis for the Global Burden of Disease Study 2021. Lancet.

[2] Okunogbe A., Nugent R., Spencer G., Powis J., Ralston J., Wilding J. (2022). Economic impacts of overweight and obesity: Current and future estimates for 161 countries. BMJ Glob. Health.

[3] Sen C.K., Gordillo G.M., Roy S., Kirsner R., Lambert L., Hunt T.K., Gottrup F., Gurtner G.C., Longaker M.T. (2009). Human skin wounds: A major and snowballing threat to public health and the economy. Wound Repair Regen..

[4] Jones R.E., Foster D.S., Longaker M.T. (2018). Management of Chronic Wounds-2018. JAMA.

[5] Le Berre C., Honap S., Peyrin-Biroulet L. (2023). Ulcerative colitis. Lancet.

[6] Kornbluth A., Sachar D.B. (2010). Ulcerative colitis practice guidelines in adults: American College Of Gastroenterology, Practice Parameters Committee. Am. J. Gastroenterol..

[7] Rodrigues M., Kosaric N., Bonham C.A., Gurtner G.C. (2019). Wound Healing: A Cellular Perspective. Physiol. Rev..

[8] Gurtner G.C., Werner S., Barrandon Y., Longaker M.T. (2008). Wound repair and regeneration. Nature.

[9] Wilkinson H.N., Hardman M.J. (2020). Wound healing: Cellular mechanisms and pathological outcomes. Open Biol..

[10] Hesketh M., Sahin K.B., West Z.E., Murray R.Z. (2017). Macrophage Phenotypes Regulate Scar Formation and Chronic Wound Healing. Int. J. Mol. Sci..

[11] Ellis S., Lin E.J., Tartar D. (2018). Immunology of Wound Healing. Curr. Dermatol. Rep..

[12] Herter E.K., Xu Landén N. (2017). Non-Coding RNAs: New Players in Skin Wound Healing. Adv. Wound Care.

[13] Veith A.P., Henderson K., Spencer A., Sligar A.D., Baker A.B. (2019). Therapeutic strategies for enhancing angiogenesis in wound healing. Adv. Drug Deliv. Rev..

[14] Basu S., Ramchuran Panray T., Bali Singh T., Gulati A.K., Shukla V.K. (2009). A prospective, descriptive study to identify the microbiological profile of chronic wounds in outpatients. Ostomy/Wound Manag..

[15] Dong Y., Wang Z. (2023). ROS-scavenging materials for skin wound healing: Advancements and applications. Front. Bioeng. Biotechnol..

[16] Gosain A., DiPietro L.A. (2004). Aging and wound healing. World J. Surg..

[17] Vileikyte L. (2007). Stress and wound healing. Clin. Dermatol..

[18] Brem H., Tomic-Canic M. (2007). Cellular and molecular basis of wound healing in diabetes. J. Clin. Investig..

[19] Wilson J.A., Clark J.J. (2004). Obesity: Impediment to postsurgical wound healing. Adv. Ski. Wound Care.

[20] Szabo G., Mandrekar P. (2009). A recent perspective on alcohol, immunity, and host defense. Alcohol. Clin. Exp. Res..

[21] Ahn C., Mulligan P., Salcido R.S. (2008). Smoking-the bane of wound healing: Biomedical interventions and social influences. Adv. Ski. Wound Care.

[22] Campos A.C., Groth A.K., Branco A.B. (2008). Assessment and nutritional aspects of wound healing. Curr. Opin. Clin. Nutr. Metab. Care.

[23] Mattei V., Santacroce C., Tasciotti V., Martellucci S., Santilli F., Manganelli V., Piccoli L., Misasi R., Sorice M., Garofalo T. (2015). Role of lipid rafts in neuronal differentiation of dental pulp-derived stem cells. Exp. Cell Res..

[24] Mattei V., Martellucci S., Pulcini F., Santilli F., Sorice M., Delle Monache S. (2021). Regenerative Potential of DPSCs and Revascularization: Direct, Paracrine or Autocrine Effect?. Stem Cell Rev. Rep..

[25] Ogata K., Moriyama M., Matsumura-Kawashima M., Kawado T., Yano A., Nakamura S. (2022). The Therapeutic Potential of Secreted Factors from Dental Pulp Stem Cells for Various Diseases. Biomedicines.

[26] Hessvik N.P., Llorente A. (2018). Current knowledge on exosome biogenesis and release. Cell. Mol. Life Sci. CMLS.

[27] Nishikawa T., Maeda K., Nakamura M., Yamamura T., Sawada T., Mizutani Y., Ito T., Ishikawa T., Furukawa K., Ohno E. (2021). Filtrated Adipose Tissue-Derived Mesenchymal Stem Cell Lysate Ameliorates Experimental Acute Colitis in Mice. Dig. Dis. Sci..

[28] Xu J., Zhang C., Yan Z., Fan C., Yuan S., Wang J., Zhu Y., Luo L., Shi K., Deng J. (2024). Dental Pulp Stem Cell Lysate-Based Hydrogel Improves Diabetic Wound Healing via the Regulation of Anti-Inflammatory Macrophages and Keratinocytes. ACS Appl. Bio Mater..

[29] Duan X., Zhang R., Feng H., Zhou H., Luo Y., Xiong W., Li J., He Y., Ye Q. (2024). A new subtype of artificial cell-derived vesicles from dental pulp stem cells with the bioequivalence and higher acquisition efficiency compared to extracellular vesicles. J. Extracell. Vesicles.

[30] Greene C.J., Anderson S., Barthels D., Howlader M.S.I., Kanji S., Sarkar J., Das H. (2022). DPSC Products Accelerate Wound Healing in Diabetic Mice through Induction of SMAD Molecules. Cells.

[31] Yang Z., Ma L., Du C., Wang J., Zhang C., Hu L., Wang S. (2023). Dental pulp stem cells accelerate wound healing through CCL2-induced M2 macrophages polarization. iScience.

[32] Anderson S., Prateeksha P., Das H. (2022). Dental Pulp-Derived Stem Cells Reduce Inflammation, Accelerate Wound Healing and Mediate M2 Polarization of Myeloid Cells. Biomedicines.

[33] Omi M., Hata M., Nakamura N., Miyabe M., Kobayashi Y., Kamiya H., Nakamura J., Ozawa S., Tanaka Y., Takebe J. (2016). Transplantation of dental pulp stem cells suppressed inflammation in sciatic nerves by promoting macrophage polarization towards anti-inflammation phenotypes and ameliorated diabetic polyneuropathy. J. Diabetes Investig..

[34] Howlader M.S.I., Prateeksha P., Hansda S., Naidu P., Das M., Barthels D., Das H. (2024). Secretory products of DPSC mitigate inflammatory effects in microglial cells by targeting MAPK pathway. Biomed. Pharmacother..

[35] Duan X., Luo Y., Zhang R., Zhou H., Xiong W., Li R., Huang Z., Luo L., Rong S., Li M. (2023). ZIF-8 as a protein delivery system enhances the application of dental pulp stem cell lysate in anti-photoaging therapy. Mater. Today Adv..

[36] Yu F.X., Lee P.S.Y., Yang L., Gao N., Zhang Y., Ljubimov A.V., Yang E., Zhou Q., Xie L. (2022). The impact of sensory neuropathy and inflammation on epithelial wound healing in diabetic corneas. Prog. Retin. Eye Res..

[37] O’Toole E.A. (2001). Extracellular matrix and keratinocyte migration. Clin. Exp. Dermatol..

[38] Matheus H.R., Hadad H., Monteiro J., Takusagawa T., Zhang F., Ye Q., He Y., Rosales I.A., Jounaidi Y., Randolph M.A. (2023). Photo-crosslinked GelMA loaded with dental pulp stem cells and VEGF to repair critical-sized soft tissue defects in rats. J. Stomatol. Oral Maxillofac. Surg..

[39] Martínez-Sarrà E., Montori S., Gil-Recio C., Núñez-Toldrà R., Costamagna D., Rotini A., Atari M., Luttun A., Sampaolesi M. (2017). Human dental pulp pluripotent-like stem cells promote wound healing and muscle regeneration. Stem Cell Res. Ther..

[40] Nyström A., Bruckner-Tuderman L. (2019). Matrix molecules and skin biology. Semin. Cell Dev. Biol..

[41] Wong T., McGrath J.A., Navsaria H. (2007). The role of fibroblasts in tissue engineering and regeneration. Br. J. Dermatol..

[42] Chin Y.T., Liu C.M., Chen T.Y., Chung Y.Y., Lin C.Y., Hsiung C.N., Jan Y.S., Chiu H.C., Fu E., Lee S.Y. (2021). 2,3,5,4′-tetrahydroxystilbene-2-O-β-D-glucoside-stimulated dental pulp stem cells-derived conditioned medium enhances cell activity and anti-inflammation. J. Dent. Sci..

[43] Zhang Y., Liu J., Zou T., Qi Y., Yi B., Dissanayaka W.L., Zhang C. (2021). DPSCs treated by TGF-β1 regulate angiogenic sprouting of three-dimensionally co-cultured HUVECs and DPSCs through VEGF-Ang-Tie2 signaling. Stem Cell Res. Ther..

[44] Zhou Z., Zheng J., Lin D., Xu R., Chen Y., Hu X. (2022). Exosomes derived from dental pulp stem cells accelerate cutaneous wound healing by enhancing angiogenesis via the Cdc42/p38 MAPK pathway. Int. J. Mol. Med..

[45] Lamalice L., Houle F., Jourdan G., Huot J. (2004). Phosphorylation of tyrosine 1214 on VEGFR2 is required for VEGF-induced activation of Cdc42 upstream of SAPK2/p38. Oncogene.

[46] Li B., Liang A., Zhou Y., Huang Y., Liao C., Zhang X., Gong Q. (2023). Hypoxia preconditioned DPSC-derived exosomes regulate angiogenesis via transferring LOXL2. Exp. Cell Res..

[47] Zhou H., Li X., Wu R.X., He X.T., An Y., Xu X.Y., Sun H.H., Wu L.A., Chen F.M. (2021). Periodontitis-compromised dental pulp stem cells secrete extracellular vesicles carrying miRNA-378a promote local angiogenesis by targeting Sufu to activate the Hedgehog/Gli1 signalling. Cell Prolif..

[48] Zhou H., Jing S., Xiong W., Zhu Y., Duan X., Li R., Peng Y., Kumeria T., He Y., Ye Q. (2023). Metal-organic framework materials promote neural differentiation of dental pulp stem cells in spinal cord injury. J. Nanobiotechnol..

[49] Chen H., Yamaguchi S., Wang Y., Kaminogo K., Sakai K., Hibi H. (2024). Cytoprotective role of human dental pulp stem cell-conditioned medium in chemotherapy-induced alopecia. Stem Cell Res. Ther..

[50] Liu D., Xu Q., Meng X., Liu X., Liu J. (2024). Status of research on the development and regeneration of hair follicles. Int. J. Med. Sci..

[51] Zanini G., Bertani G., Di Tinco R., Pisciotta A., Bertoni L., Selleri V., Generali L., Marconi A., Mattioli A.V., Pinti M. (2024). Dental Pulp Stem Cells Modulate Inflammasome Pathway and Collagen Deposition of Dermal Fibroblasts. Cells.

[52] Dai J., Shen J., Chai Y., Chen H. (2021). IL-1β Impaired Diabetic Wound Healing by Regulating MMP-2 and MMP-9 through the p38 Pathway. Mediat. Inflamm..

[53] Wu X., Yang L., Zheng Z., Li Z., Shi J., Li Y., Han S., Gao J., Tang C., Su L. (2016). Src promotes cutaneous wound healing by regulating MMP-2 through the ERK pathway. Int. J. Mol. Med..

[54] Kanji S., Sarkar R., Pramanik A., Kshirsagar S., Greene C.J., Das H. (2021). Dental pulp-derived stem cells inhibit osteoclast differentiation by secreting osteoprotegerin and deactivating AKT signalling in myeloid cells. J. Cell. Mol. Med..

[55] Lo Monaco M., Gervois P., Beaumont J., Clegg P., Bronckaers A., Vandeweerd J.M., Lambrichts I. (2020). Therapeutic Potential of Dental Pulp Stem Cells and Leukocyte- and Platelet-Rich Fibrin for Osteoarthritis. Cells.

[56] Zhao X., Chen J., Sun H., Zhang Y., Zou D. (2022). New insights into fibrosis from the ECM degradation perspective: The macrophage-MMP-ECM interaction. Cell Biosci..

[57] O’Rourke S.A., Dunne A., Monaghan M.G. (2019). The Role of Macrophages in the Infarcted Myocardium: Orchestrators of ECM Remodeling. Front. Cardiovasc. Med..

[58] Sonkar V.K., Eustes A.S., Ahmed A., Jensen M., Solanki M.V., Swamy J., Kumar R., Fidler T.P., Houtman J.C.D., Allen B.G. (2023). Endogenous SOD2 (Superoxide Dismutase) Regulates Platelet-Dependent Thrombin Generation and Thrombosis During Aging. Arterioscler. Thromb. Vasc. Biol..

[59] Wang G., Yang F., Zhou W., Xiao N., Luo M., Tang Z. (2023). The initiation of oxidative stress and therapeutic strategies in wound healing. Biomed. Pharmacother..

[60] Deng L., Du C., Song P., Chen T., Rui S., Armstrong D.G., Deng W. (2021). The Role of Oxidative Stress and Antioxidants in Diabetic Wound Healing. Oxid. Med. Cell. Longev..

[61] Guan Y., Niu H., Liu Z., Dang Y., Shen J., Zayed M., Ma L., Guan J. (2021). Sustained oxygenation accelerates diabetic wound healing by promoting epithelialization and angiogenesis and decreasing inflammation. Sci. Adv..

[62] Mishra M., Raik S., Rattan V., Bhattacharyya S. (2023). Mitochondria transfer as a potential therapeutic mechanism in Alzheimer’s disease-like pathology. Brain Res..

[63] Zielińska M., Pawłowska A., Orzeł A., Sulej L., Muzyka-Placzyńska K., Baran A., Filipecka-Tyczka D., Pawłowska P., Nowińska A., Bogusławska J. (2023). Wound Microbiota and Its Impact on Wound Healing. Int. J. Mol. Sci..

[64] Canesso M.C., Vieira A.T., Castro T.B., Schirmer B.G., Cisalpino D., Martins F.S., Rachid M.A., Nicoli J.R., Teixeira M.M., Barcelos L.S. (2014). Skin wound healing is accelerated and scarless in the absence of commensal microbiota. J. Immunol..

[65] Afami M.E., El Karim I., About I., Krasnodembskaya A.D., Laverty G., Lundy F.T. (2021). Multicomponent Peptide Hydrogels as an Innovative Platform for Cell-Based Tissue Engineering in the Dental Pulp. Pharmaceutics.

[66] Marrelli M., Codispoti B., Shelton R.M., Scheven B.A., Cooper P.R., Tatullo M., Paduano F. (2018). Dental Pulp Stem Cell Mechanoresponsiveness: Effects of Mechanical Stimuli on Dental Pulp Stem Cell Behavior. Front. Physiol..

[67] Zhou S., Xie M., Su J., Cai B., Li J., Zhang K. (2023). New insights into balancing wound healing and scarless skin repair. J. Tissue Eng..

[68] Mascharak S., desJardins-Park H.E., Davitt M.F., Griffin M., Borrelli M.R., Moore A.L., Chen K., Duoto B., Chinta M., Foster D.S. (2021). Preventing Engrailed-1 activation in fibroblasts yields wound regeneration without scarring. Science.

[69] McWhorter F.Y., Wang T., Nguyen P., Chung T., Liu W.F. (2013). Modulation of macrophage phenotype by cell shape. Proc. Natl. Acad. Sci. USA.

[70] Brown I.A.M., Diederich L., Good M.E., DeLalio L.J., Murphy S.A., Cortese-Krott M.M., Hall J.L., Le T.H., Isakson B.E. (2018). Vascular Smooth Muscle Remodeling in Conductive and Resistance Arteries in Hypertension. Arterioscler. Thromb. Vasc. Biol..

[71] Hess J., Barysch-Bonderer M.J., Seeli C., Laube J., Ghosh A., Deinsberger J., Weber B., Hafner J., Meier-Schiesser B. (2024). Identifying Key Drivers in the Pathogenesis of Martorell Hypertensive Ischaemic Leg Ulcer: A Comparative Analysis with Chronic Venous Leg Ulcer. Acta Derm.-Venereol..

[72] Ma J., Li Y., Yang X., Liu K., Zhang X., Zuo X., Ye R., Wang Z., Shi R., Meng Q. (2023). Signaling pathways in vascular function and hypertension: Molecular mechanisms and therapeutic interventions. Signal Transduct. Target. Ther..

[73] Arima J., Huang C., Rosner B., Akaishi S., Ogawa R. (2015). Hypertension: A systemic key to understanding local keloid severity. Wound Repair Regen..

[74] Yafi F.A., Jenkins L., Albersen M., Corona G., Isidori A.M., Goldfarb S., Maggi M., Nelson C.J., Parish S., Salonia A. (2016). Erectile dysfunction. Nat. Rev. Dis. Primers.

[75] Koga S., Horiguchi Y. (2022). Efficacy of a cultured conditioned medium of exfoliated deciduous dental pulp stem cells in erectile dysfunction patients. J. Cell. Mol. Med..

[76] Diomede F., Guarnieri S., Lanuti P., Konstantinidou F., Gatta V., Rajan T.S., Pierdomenico S.D., Trubiani O., Marconi G.D., Pizzicannella J. (2024). Extracellular vesicles (EVs): A promising therapeutic tool in the heart tissue regeneration. BioFactors.

[77] Rajendran S.B., Challen K., Wright K.L., Hardy J.G. (2021). Electrical Stimulation to Enhance Wound Healing. J. Funct. Biomater..

[78] Sun J., Xu C., Wo K., Wang Y., Zhang J., Lei H., Wang X., Shi Y., Fan W., Zhao B. (2024). Wireless Electric Cues Mediate Autologous DPSC-Loaded Conductive Hydrogel Microspheres to Engineer the Immuno-Angiogenic Niche for Homologous Maxillofacial Bone Regeneration. Adv. Healthc. Mater..

[79] Gu Y., Zhang W., Wu X., Zhang Y., Xu K., Su J. (2023). Organoid assessment technologies. Clin. Transl. Med..

[80] Teles E.S.A.L., Yokota-Moreno B.Y., Branquinho M.S., Salles G.R., de Souza T.C., de Carvalho R.A., Batista G., Varella Branco E., Griesi-Oliveira K., Passos Bueno M.R. (2024). Generation and characterization of cortical organoids from iPSC-derived dental pulp stem cells using traditional and innovative approaches. Neurochem. Int..

[81] Tatullo M., Cocco T., Ferretta A., Caroppo R., Marrelli B., Spagnuolo G., Paduano F. (2024). Unveiling the Neurodegenerative Alterations through Oral Stem Cells. J. Dent. Res..

[82] Liu F., Xiao J., Chen L.H., Pan Y.Y., Tian J.Z., Zhang Z.R., Bai X.C. (2024). Self-assembly of differentiated dental pulp stem cells facilitates spheroid human dental organoid formation and prevascularization. World J. Stem Cells.

[83] Williams D.W., Wu H., Oh J.E., Fakhar C., Kang M.K., Shin K.H., Park N.H., Kim R.H. (2013). 2-Hydroxyethyl methacrylate inhibits migration of dental pulp stem cells. J. Endod..

[84] Khayat A., Monteiro N., Smith E.E., Pagni S., Zhang W., Khademhosseini A., Yelick P.C. (2017). GelMA-Encapsulated hDPSCs and HUVECs for Dental Pulp Regeneration. J. Dent. Res..

[85] Badhe R.V., Godse A., Shinkar A., Kharat A., Patil V., Gupta A., Kheur S. (2021). Development and Characterization of Conducting-Polymer-Based Hydrogel Dressing for Wound Healing. Turk. J. Pharm. Sci..

[86] Dasgupta S., Reddy K.P., Datta P., Barui A. (2023). Vitamin D3-incorporated chitosan/collagen/fibrinogen scaffolds promote angiogenesis and endothelial transition via HIF-1/IGF-1/VEGF pathways in dental pulp stem cells. Int. J. Biol. Macromol..

[87] Lu W., Zhao J., Cai X., Wang Y., Lin W., Fang Y., Wang Y., Ao J., Shou J., Xu J. (2024). Cadherin-responsive hydrogel combined with dental pulp stem cells and fibroblast growth factor 21 promotes diabetic scald repair via regulating epithelial-mesenchymal transition and necroptosis. Mater. Today Bio.

[88] avydova G.A., Chaikov L.L., Melnik N.N., Gainutdinov R.V., Selezneva I.I., Perevedentseva E.V., Mahamadiev M.T., Proskurin V.A., Yakovsky D.S., Mohan A.G. (2024). Polysaccharide Composite Alginate-Pectin Hydrogels as a Basis for Developing Wound Healing Materials. Polymers.

[89] Oudhoff M.J., van den Keijbus P.A., Kroeze K.L., Nazmi K., Gibbs S., Bolscher J.G., Veerman E.C. (2009). Histatins enhance wound closure with oral and non-oral cells. J. Dent. Res..

[90] Frank S., Stallmeyer B., Kämpfer H., Kolb N., Pfeilschifter J. (2000). Leptin enhances wound re-epithelialization and constitutes a direct function of leptin in skin repair. J. Clin. Investig..

[91] Ren L., Jiang Z., Zhang H., Chen Y., Zhu D., He J., Chen Y., Wang Y., Yang G. (2023). Biomaterials derived from hard palate mucosa for tissue engineering and regenerative medicine. Mater. Today Bio.

[92] Shen P., Ma Z., Xu X., Li W., Li Y. (2024). Dental pulp stem cells promote malignant transformation of oral epithelial cells through mitochondrial transfer. Med. Mol. Morphol..

[93] Salkin H., Acar M.B., Korkmaz S., Gunaydin Z., Gonen Z.B., Basaran K.E., Ozcan S. (2022). Transforming growth factor β1-enriched secretome up-regulate osteogenic differentiation of dental pulp stem cells, and a potential therapeutic for gingival wound healing: A comparative proteomics study. J. Dent..

[94] Forkel S., Schubert S., Corvin L., Heine G., Lang C.C.V., Oppel E., Pföhler C., Treudler R., Bauer A., Sulk M. (2024). Contact allergies to dental materials in patients. Br. J. Dermatol..

[95] Chen L., Tong Z., Luo H., Qu Y., Gu X., Si M. (2023). Titanium particles in peri-implantitis: Distribution, pathogenesis and prospects. Int. J. Oral Sci..

[96] Eren Belgin E., Genç D., Tekin L., Sezgin S., Aladağ A. (2024). Anti-Inflammatory Effect of Dental Pulpa Mesenchymal Stem Cell Exosomes Loaded Mucoadhesive Hydrogel on Mice with Dental Nickel Hypersensitivity. Macromol. Biosci..

[97] Han B., Zheng R., Zeng H., Wang S., Sun K., Chen R., Li L., Wei W., He J. (2024). Cancer incidence and mortality in China, 2022. J. Natl. Cancer Cent..

[98] Yang H., Wang F., Hallemeier C.L., Lerut T., Fu J. (2024). Oesophageal cancer. Lancet.

[99] Zhang C., Zhang Y., Feng Z., Zhang F., Liu Z., Sun X., Ruan M., Liu M., Jin S. (2018). Therapeutic effect of dental pulp stem cell transplantation on a rat model of radioactivity-induced esophageal injury. Cell Death Dis..

[100] He X.W., He X.S., Lian L., Wu X.J., Lan P. (2012). Systemic infusion of bone marrow-derived mesenchymal stem cells for treatment of experimental colitis in mice. Dig. Dis. Sci..

[101] Song W.J., Li Q., Ryu M.O., Ahn J.O., Bhang D.H., Jung Y.C., Youn H.Y. (2018). TSG-6 released from intraperitoneally injected canine adipose tissue-derived mesenchymal stem cells ameliorate inflammatory bowel disease by inducing M2 macrophage switch in mice. Stem Cell Res. Ther..

[102] Lin Y., Lin L., Wang Q., Jin Y., Zhang Y., Cao Y., Zheng C. (2015). Transplantation of human umbilical mesenchymal stem cells attenuates dextran sulfate sodium-induced colitis in mice. Clin. Exp. Pharmacol. Physiol..

[103] Yui S., Nakamura T., Sato T., Nemoto Y., Mizutani T., Zheng X., Ichinose S., Nagaishi T., Okamoto R., Tsuchiya K. (2012). Functional engraftment of colon epithelium expanded in vitro from a single adult Lgr5⁺ stem cell. Nat. Med..

[104] Zheng L., Duan S.L. (2023). Molecular regulation mechanism of intestinal stem cells in mucosal injury and repair in ulcerative colitis. World J. Gastroenterol..

[105] Ma Y., Lang X., Yang Q., Han Y., Kang X., Long R., Du J., Zhao M., Liu L., Li P. (2023). Paeoniflorin promotes intestinal stem cell-mediated epithelial regeneration and repair via PI3K-AKT-mTOR signalling in ulcerative colitis. Int. Immunopharmacol..

[106] Xu J., Wang X., Chen J., Chen S., Li Z., Liu H., Bai Y., Zhi F. (2020). Embryonic stem cell-derived mesenchymal stem cells promote colon epithelial integrity and regeneration by elevating circulating IGF-1 in colitis mice. Theranostics.

[107] Miyamoto S., Ohnishi S., Onishi R., Tsuchiya I., Hosono H., Katsurada T., Yamahara K., Takeda H., Sakamoto N. (2017). Therapeutic effects of human amnion-derived mesenchymal stem cell transplantation and conditioned medium enema in rats with trinitrobenzene sulfonic acid-induced colitis. Am. J. Transl. Res..

[108] Li N., Zhang Y., Nepal N., Li G., Yang N., Chen H., Lin Q., Ji X., Zhang S., Jin S. (2021). Dental pulp stem cells overexpressing hepatocyte growth factor facilitate the repair of DSS-induced ulcerative colitis. Stem Cell Res. Ther..

[109] Zhao Y., Wang L., Jin Y., Shi S. (2012). Fas ligand regulates the immunomodulatory properties of dental pulp stem cells. J. Dent. Res..

[110] Aly R.M., Abohashem R.S., Ahmed H.H., Halim A.S.A. (2024). Combinatorial intervention with dental pulp stem cells and sulfasalazine in a rat model of ulcerative colitis. Inflammopharmacology.

[111] Gonullu D.C., Huang X.M., Robinson L.G., Walker C.A., Ayoola-Adeola M., Jameson R., Yim D., Awonuga A. (2022). Tubal factor infertility and its impact on reproductive freedom of African American women. Am. J. Obstet. Gynecol..

[112] Li Z., Zhang Z., Ming W.K., Chen X., Xiao X.M. (2017). Tracing GFP-labeled WJMSCs in vivo using a chronic salpingitis model: An animal experiment. Stem Cell Res. Ther..

[113] Tuddenham S., Hamill M.M., Ghanem K.G. (2022). Diagnosis and Treatment of Sexually Transmitted Infections: A Review. JAMA.

[114] Weiss N.S., Kostova E., Nahuis M., Mol B.W.J., van der Veen F., van Wely M. (2019). Gonadotrophins for ovulation induction in women with polycystic ovary syndrome. Cochrane Database Syst. Rev..

[115] Bouyer J., Job-Spira N., Pouly J.L., Coste J., Germain E., Fernandez H. (2000). Fertility following radical, conservative-surgical or medical treatment for tubal pregnancy: A population-based study. BJOG Int. J. Obstet. Gynaecol..

[116] Akash M.S., Rehman K., Sun H., Chen S. (2013). Sustained delivery of IL-1Ra from PF127-gel reduces hyperglycemia in diabetic GK-rats. PLoS ONE.

[117] Youn J., Choi J.H., Lee S., Lee S.W., Moon B.K., Song J.E., Khang G. (2021). Pluronic F-127/Silk Fibroin for Enhanced Mechanical Property and Sustained Release Drug for Tissue Engineering Biomaterial. Materials.

[118] Albashari A., He Y., Zhang Y., Ali J., Lin F., Zheng Z., Zhang K., Cao Y., Xu C., Luo L. (2020). Thermosensitive bFGF-Modified Hydrogel with Dental Pulp Stem Cells on Neuroinflammation of Spinal Cord Injury. ACS Omega.

[119] Luo L., Zhu Q., Li Y., Hu F., Yu J., Liao X., Xing Z., He Y., Ye Q. (2022). Application of thermosensitive-hydrogel combined with dental pulp stem cells on the injured fallopian tube mucosa in an animal model. Front. Bioeng. Biotechnol..

[120] Toma A.I., Fuller J.M., Willett N.J., Goudy S.L. (2021). Oral wound healing models and emerging regenerative therapies. Transl. Res. J. Lab. Clin. Med..

[121] Rowlatt U. (1979). Intrauterine wound healing in a 20 week human fetus. Virchows Arch. A.

[122] Monavarian M., Kader S., Moeinzadeh S., Jabbari E. (2019). Regenerative Scar-Free Skin Wound Healing. Tissue Eng. Part B Rev..

[123] Moon J., Yoon J.Y., Yang J.H., Kwon H.H., Min S., Suh D.H. (2019). Atrophic acne scar: A process from altered metabolism of elastic fibres and collagen fibres based on transforming growth factor-β1 signalling. Br. J. Dermatol..

[124] McCollum P.T., Bush J.A., James G., Mason T., O’Kane S., McCollum C., Krievins D., Shiralkar S., Ferguson M.W. (2011). Randomized phase II clinical trial of avotermin versus placebo for scar improvement. Br. J. Surg..

